# Severe Undifferentiated Vasoplegic Shock Refractory to Vasoactive Agents Treated with Methylene Blue

**DOI:** 10.1155/2017/8747326

**Published:** 2017-10-02

**Authors:** Farheen Manji, Benjamin Wierstra, Juan Posadas

**Affiliations:** ^1^University of Calgary and Alberta Health Services, Calgary, AB, Canada; ^2^Department of Medicine, University of Calgary, Calgary, AB, Canada; ^3^Department of Critical Care, University of Calgary, Calgary, AB, Canada

## Abstract

Methylene blue is a phenothiazine-related heterocyclic aromatic molecule presently used in the treatment of methemoglobinemia. Recently, it has been implicated in the treatment of severe refractory vasoplegic shock caused by anaphylaxis, sepsis, or postcardiopulmonary bypass. We present a case of a 27-year-old male with profound vasoplegic shock of unknown etiology which was refractory to vasopressors who responded within hours to a single dose of methylene blue. Additionally, we review the evidence of methylene blue's role in the treatment of shock. This case illustrates a diagnostic approach and treatment options in the setting of undifferentiated vasodilatory shock and outlines a new and emerging role for methylene blue in this clinical setting.

## 1. Introduction

Vasodilatory shock is a state of hypoperfusion characterized by a significant decrease of vasomotor tone and consequently a decrease of systemic vascular resistance [1]. There are many prevailing theories regarding the pathophysiology of vasodilation that center around the inappropriate activation of vasodilator mechanisms and the dysregulation of vasoconstriction. One such mechanism involves excessive production of nitric oxide (NO) and upregulation of cyclic guanosine 3′,5′-monophosphate (cGMP) which results in dephosphorylation of myosin and subsequent vasodilation [1]. Vasodilatory shock has multiple etiologies including sepsis, anaphylaxis, adrenal insufficiency, drug-induced shock, and postcardiopulmonary bypass vasoplegia [1]. Treatment usually includes addressing the underlying cause and the provision of supportive care including intravenous fluids and vasoactive agents [2]. In addition to conventional therapies, there has been research into inhibiting the cellular pathways at the level of NO and cGMP to prevent vasodilation and tissue hypoperfusion [2]. One such therapy for refractory vasodilatory shock is methylene blue which is a guanylyl cyclase inhibitor and inhibits production of cGMP and therefore inhibits dephosphorylation of myosin, decreasing vasodilation [1].

We present the case of a 27-year-old male with profound vasoplegic shock refractory to vasoactive agents in the setting of intravenous methamphetamine use who responded within hours to methylene blue therapy. Additionally, we review the current literature on the use of methylene blue in the setting of distributive shock of various etiologies including anaphylaxis, sepsis, and postcardiac bypass.

## 2. Case Presentation

A 27-year-old with past medical history of polysubstance abuse, including methamphetamines, cocaine, opioids, and alcohol, presented to the emergency department after two days of intravenous methamphetamine use with generalized malaise, nausea, dizziness, and blurry vision. He denied fever, chills, chest pain, or shortness of breath. He had no recent sick contacts. He purchased his methamphetamines from his regular dealer but did admit to feeling unwell a few hours after injecting it.

His vitals on arrival to the emergency department were a temperature of 36.1 Celsius, heart rate of 122 bpm, blood pressure of 80/60 mmHg with O2 saturation of 92% on room air. On examination, his extremities were warm. He was alert and oriented to person, place, and time. His neurological exam revealed no motor or sensory deficits. His neck was supple. His cardiovascular exam revealed a regular tachycardia but no murmur. His respiratory exam was unremarkable. His abdomen was mildly tender in the right upper quadrant.

His initial laboratory work is shown in Table 1. In summary, he presented with a mild normocytic anemia, new thrombocytopenia, and new renal failure with a urinalysis showing no casts with trace blood and protein. He had coagulation abnormalities consistent with disseminated intravascular coagulation (DIC). His initial arterial blood gas showed a primary metabolic acidosis secondary to lactic acidosis with a partially compensated by a respiratory alkalosis. Computed Tomography (CT) scan of his head, chest, abdomen, and pelvis was only significant for mild atelectasis in the posterior aspect of both lower lobes and splenomegaly (measured at 15.8 cm, normal < 12 cm). An echocardiogram revealed normal right and left ventricular function with no hemodynamically significant valvular disease. One out of three sets of blood cultures was positive for coryneform bacilli at 65 hours which was thought to be a contaminant. HIV and hepatitis serologies were negative.

**Figure 1:**
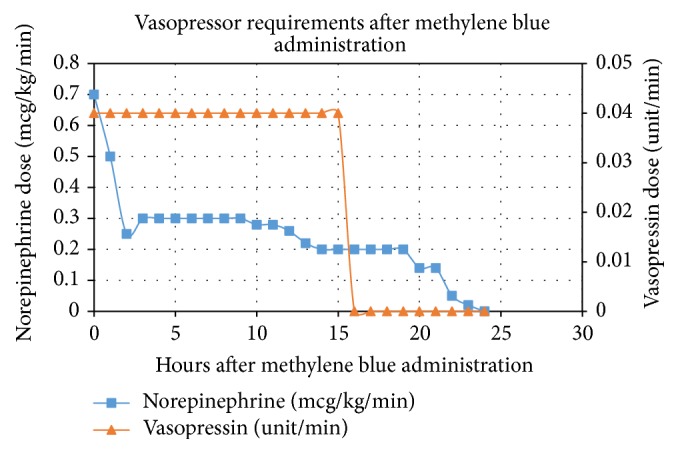
Vasopressor requirements hours after methylene blue administration.

In the emergency department, his blood pressure declined to 62/34 mmHg. He was given three liters of normal saline initially with no response in blood pressure. He was then started on escalating doses of norepinephrine up to 0.9 mcg/kg/min after which his blood pressure increased to 70/40 mmHg. Epinephrine was then added at 0.2 mcg/kg/min, resulting in his blood pressure increasing to 100/50 mmHg. He was empirically started on piperacillin-tazobactam and vancomycin. Given that he had no biochemical evidence of adrenal insufficiency, steroids were not given. Upon transfer to the ICU, the patient was intubated and his hemodynamic profile remained tenuous with his blood pressure dropping to 74/26 mmHg. His norepinephrine was increased to 1 mcg/kg/min, epinephrine was increased to 0.35 mcg/kg/min, and vasopressin was added at 0.04 unit/min. Forty-eight hours into his admission, his lactate normalized but he was still requiring 0.9 mcg/kg/min of norepinephrine and 0.04 unit/min of vasopressin to maintain a mean arterial pressure greater than 60 mmHg. He was given methylene blue dosed at 2 mg/kg for a total dose of 190 mg once. His blood pressure and vasoactive agent requirements are illustrated in Figure 1 in relation to the number of hours after methylene blue administration. Within two hours of administration, the norepinephrine dosage was halved, within 15 hours, he was weaned off vasopressin and within 24 hours, he was off vasoactive support completely and successfully extubated.

No septic or anaphylactic etiology was identified as a cause of the patient's distributive shock. There was no clinical or biochemical evidence of adrenal insufficiency. He denied any overdoses of medications known to commonly cause vasoplegic shock such as calcium channel blockers. As such, the etiology of his shock remained undifferentiated.

## 3. Discussion

**Figure 2:**
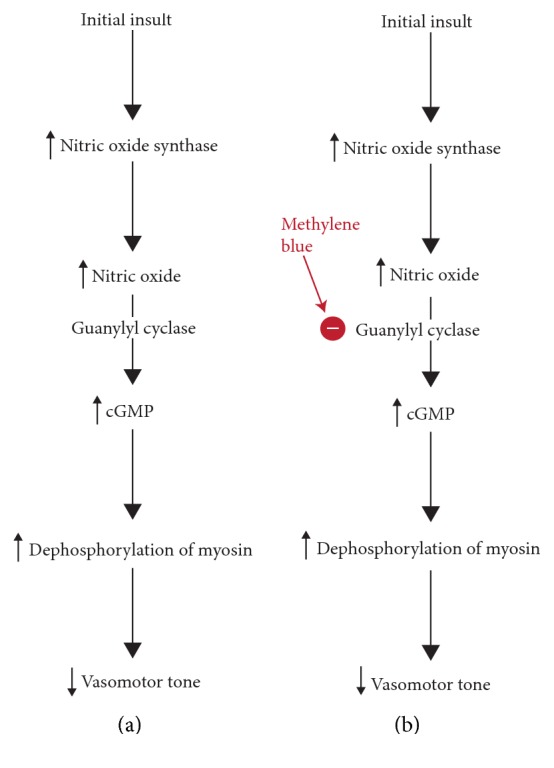
(a) The biochemical pathway of vasodilatory shock as a result of upregulation of the nitric oxide-cGMP pathway. (b) Methylene blue's site of action, as a guanylyl cyclase inhibitor, resulting in increased vasomotor tone.

Methylene blue is a phenothiazine-related heterocyclic aromatic molecule that has a long history of use, dating back to the 1800s for treatment of malaria and more commonly, methemoglobinemia [3]. Recently, there has been emerging use of methylene blue in the treatment of refractory distributive shock via inhibition of the nitric-oxide cyclic guanosine monophosphate pathway (Figure 2) [1]. An initial insult results in an increase in nitric oxide synthase and nitric oxide which, via guanylyl cyclase, increases cGMP, and downstream signaling, ultimately resulting in dephosphorylation of myosin and vasodilation [1].

Various in vitro and in vivo animal studies have shown methylene blue as a selective inhibitor of cGMP, counteracting its downstream vasodilatory effects in shock [3]. Clinically, the most well-established use of methylene blue in shock is with patients after cardiac bypass surgery with a range of observational data as well as randomized controlled trials [4]. While not proven as a first line agent, it has been shown to improve systemic vascular resistance if therapy with norepinephrine fails [4]. There have been case reports and series demonstrating the efficacy of methylene blue in cases of anaphylactic shock with resolution of hypotension within one hour of administration [3]. In septic shock, several small randomized control trials support the use of methylene blue, suggesting improved hemodynamics through increase in mean arterial pressure and systemic vascular resistance but no mortality benefit, likely a factor of the small sample sizes [5]. The use of methylene blue in drug-induced vasoplegic shock has very little data to corroborate its regular use; some observational studies have shown a benefit in hemodynamics while others have showed no change. A recent systematic analysis reviewed 17 cases of drug-induced shock described in the literature, most commonly as a result of calcium channel blocker overdose, which showed that there was varying evidence for the use, efficacy, and dosing of methylene blue [6].

There are various dosing regimens for treating shock with methylene blue. Overall experimental and clinical data suggest that 1-2 mg/kg as a single one-time dose is effective [3]. There are other regimens which include continuous infusions and repeat boluses but there is no evidence suggesting that those are more efficacious. Consideration must also be taken of the side effect profile for methylene blue. It can commonly cause dizziness, tremors, nausea, vomiting, and discoloration of bodily fluids [3]. Less commonly, it can cause an acute hemolytic anemia or precipitation of serotonin syndrome [3]. Higher doses of methylene blue are associated with more serious adverse effects [3].

Our patient presented with severe undifferentiated vasoplegic shock that was not of septic or anaphylactic etiology and was refractory to vasoactive agents. Based on a literature search, there is minimal evidence for use of methylene blue in patients who present with a vasoplegic syndrome that is not postcardiac bypass or due to sepsis or anaphylaxis. There are no case reports or observational studies describing the use of methylene blue in undifferentiated vasoplegic shock. The patient's hemodynamics improved within hours of administration of methylene blue and he was completely off vasoactive support within twenty-four hours (Figure 1). This case report further adds to the growing body of literature that methylene blue has a significant role in the treatment of vasoplegic shock but additionally highlights its effects on shock that is of an unclear etiology, having ruled out sepsis, anaphylaxis, and adrenal insufficiency.

## 4. Conclusion

Methylene blue has growing evidence of its use as an adjunctive therapy in refractory vasoplegic shock caused after cardiac bypass or by sepsis or anaphylactic shock. This case report suggests that, in patients with severe undifferentiated vasoplegic shock that is unresponsive to vasoactive agents, there is potentially a role for use of a single dose of methylene blue as a rescue therapy.

## Figures and Tables

**Table 1:** Bloodwork on initial presentation to the emergency department.

	At admission	Normal range
Hemoglobin	128	137–180 g/L
White blood cell	8.6	4.0–11.0 × 10^9^/L
Platelets	66	150–400 × 10^9^/L
Sodium	129	133–145 mmol/L
Potassium	3.7	3.3–5.1 mmol/L
Chloride	94	98–111 mmol/L
Bicarbonate	15	21–31 mmol/L
Creatinine	418	50–120 *µ*mol/L
Total bilirubin	47	0–24 *µ*mol/L
INR	2.2	0.9–1.1
PTT	39.2	27–37 s
Lactate dehydrogenase (LDH)	377	100–235 U/L
Ferritin	754	30–400 *µ*g/L
D-Dimer	>10	<0.46 mg/L
Fibrinogen	1.1	1.6–4.1 g/L
Creatinine kinase	929	0–195 U/L
Lactate	8.0	<2 mmol/L
Random cortisol	626	nmol/L
Arterial pH	7.20	7.35–7.45
Arterial pCO2	21	35–45 mmHg
Arterial pO2	61	80–100 mmHg
Arterial bicarbonate	9	24–26 mmgHg
